# Practical Notes and Formulæ

**Published:** 1883-09-20

**Authors:** 


### PRACTICAL NOTES AND FORMULÆ.

**Hypophosphite of Lime in Cancer.**—Dr. J. B. Johnson, in Medical and Surgical Reporter, says: Some time ago I received a copy of a lecture by Dr. Hunter McGuire, of Richmond, Va., on the subject of "Cancer of the Breast," in which he recommended the use of hypophosphite of lime and soda. His formula is:

| | | |
|---|---|---|
| ℞ | Hypophosphite of lime and soda | ℥ ss, |
| | Diluted phosphoric acid | ʒ ss, |
| | Distilled water | ℥ viij. |

M. Sig. Teaspoonful in water three times a day, and when indicatedf he sometimes uses in addition, arsenic and iron in the forms of chlorides of arsenic and iron.

At the time of reading the lecture I had under my care two cases of cancer, one of the breast and one of the ear, at the angle ot the left jaw. About a year before I was consulted in the case of cancer of the breast; the breast had been entirely excised; but the wound made no effort to heal, and grew to be an ulcer two inches wide by two inches long. The cancer of the ear also presented an ulcer, irregular in shape, covering the space of an inch or more in extent. I gave at once internally—

| | | |
|---|---|---|
| ℞ | Hypophosphite of lime | ʒ iss, |
| | Bromide of potassium | ʒ ij, |
| | Fowler's solution | ʒ iss, |
| | Aqua destil | ʒ viij. |

M. Sig. Dose, a tablespoonful every three hours.

| | | |
|---|---|---|
| R | Tar | aa. ℥ j. |
| | Alcohol | |

M. Sig. Apply freely to the ulcers three times a day.

Both patients have been using the above prescription for six months, and the progress of the cancers is not only arrested, but the ulcers almost healed. There is no doubt that the progress of the cancer can be delayed by the use of the hypophosphites in combination with arsenic.

**For Torpid Liver.**—The following is suggested by Professor Delafield, of New York:

| | | |
|---|---|---|
| ℞ | Podophyllin | gr. 2, |
| | Hydrarg bichlorid | gr. 1, |
| | Pulv. ipecac | gr. 4, |
| | Ext. colocynth co | gr. 10. |

M. Ft. pil. No. 20.

I would give him a pill composed of these ingredients in the above propoations, and let him begin by taking three such pills each day. He may then gradually lessen the number as his symptoms improve.—*Gazette.*

**Naphthol for Local Sweating.**—From the New Orleans Medical and Surgical Journal, for June, we note that the Repertorie de Pharmacie gives the following formula for hyperidrosis, or excessive sweating of the hands, feet and axilla:

| | |
|---|---|
| Naphthol | 5 parts, |
| Glycerine | 10 parts, |
| Alcohol | 100 parts. |

To be used as a lotion twice a day, afterwards dusting the parts with pulverized starch or with a powder made as follows:

| | |
|---|---|
| Pulverized naphthol | 2 parts, |
| Starch | 100 parts. |

For perspiration of the feet, a pledget of cotton impregnated with the above powder is placed between the toes.—*Journal de Medecine et de Chirurgie Pratique.*

**"Pain-Killers."**—"New Idea" gives the composition of two of these nostrums, as follows:

1. Richter's Pain Expeller.

From 100 parts of capsicum make 600 parts of tincture; add a solution of 22 parts of soap in 100 of water; add thereto:

| | |
|---|---|
| Water of ammonia | 300 parts, |
| Camphor | 30 " |
| Oil of rosemary | 10 " |
| Oil of lavender | 10 " |
| Oil of thyme | 10 " |
| Oil of cloves | 10 " |
| Oil of cinnamon | 1½ " |
| Sugar coloring | q. s. |

Mix and filter.

2. Perry Davis' Pain Killer.

| | |
|---|---|
| Myrrh | 1¼ ℔s, |
| Capsicum | 10 ozs. |
| Opium | 8 " |
| Benzoin | 6 " |
| Guaiac | 3 " |
| Camphor | 10 " |
| Alcohol | 5 gals. |

—*New Remedies.*

**For Excoriatde Nipples.**—As an application to excoriated nipples the following is recommended:

| | |
|---|---|
| ℞ Balsam Peru | ʒj, |
| Olei | ʒjss, |
| Aquæ | ℥j, |
| Mucil. acaciæ | ʒjss. |

M. Sig. Apply after last nursing, the nipples having been carefully cleansed.—*Ex*

**Eczema of the Hands.**—Dr. Van Harlingen in Philadelphia Medical Times, says: Many cases of acute eczema of the hands get well under the use of a saturated solution of boracic acid, and this application is particularly useful where there are numerous vesicular lesions inclining to coalesce and break down into eczema rubrum.

In such forms of the disease it is also that the old and tried calamine and zinc wash frequently proves efficacious. It is composed as follows:

℞ Pulv. calaminis præp. . . . . . . . . . . . . . . . . . . . . . . ʒ i,

Pulv. zinci oxidi. . . . . . . . . . . . . . . . . . . . . . . . . ʒ i–ʒ ii,

Glycerinæ. . . . . . . . . . . . . . . . . . . . . . . . . . . . . . ʒ iii,

Aq. rosæ. . . . . . . . . . . . . . . . . . . . . . . . . . . . . . . ℥ iv.

M. I have recently used with advantage a solution of sulphate of zinc in water:

℞ Zinci sulphat. . . . . . . . . . . . . . . . . . . . . . . . . . . . . ʒ ss,

Aquæ. . . . . . . . . . . . . . . . . . . . . . . . . . . . . . . . . . . O i. M.

Also ointments of oleate of zinc or oleate of bismuth may be of service in some cases of acute and subacute eczema. The ointment of oleate of bismuth is most conveniently prescribed according to the following formula:

℞ Bismuthi oxidi . . . . . . . . . . . . . . . . . . . . . . . . . . . . ʒ i,

Acid oleici. . . . . . . . . . . . . . . . . . . . . . . . . . . . . . . ℥ i,

Ceræ albæ . . . . . . . . . . . . . . . . . . . . . . . . . . . . . . . ʒ iii,

Vaselini. . . . . . . . . . . . . . . . . . . . . . . . . . . . . . . . . ʒ ix,

Ol. rosæ. . . . . . . . . . . . . . . . . . . . . . . . . . . . . . . . . *m* ii.

M. This very elegant pharmaceutical preparation was first suggested by Dr. McCall Anderson, several years ago, and it is a most useful remedy in eczema of whatever locality, but its action is particularly satisfactory in eczema of the hands.

One which I have employed in many cases with most satisfactory results is the ointment of calomel and zinc:

℞ Hydrarg. chlor. mite . . . . . . . . . . . . . . . . . . . gr. x-xxx,
Ung. zinci oxidi . . . . . . . . . . . . . . . . . . . . . . . ℥ i. M.

**Remedy for Migraine, or Hemicrania.**—The following is Dr. Hermann Hager's Pulvis anti-hemicranicus imperialis—

℞ Quinidæ sulphatis. . . . . . . . . . . . . . 1.50 gm.= 24 grains,

Caffeinæ. . . . . . . . . . . . . . . . . . . . . . . 1.00 gm.= 15 "

Acidi tartarici. . . . . . . . . . . . . . . . . 1.00 gm.= 16 "

Morphiæ puræ. . . . . . . . . . . . . . . . .05 gm.= ¾ "

Sacchari albi. . . . . . . . . . . . . . . . . 10.00 gm.=150 "

Mix, and make into five powders.

One of these is to be taken mornings and evenings. Said to be a sure remedy in hemicrania. If necessary, the quantity of morphia may be slightly increased. Feeble persons should divide each powder in two parts, and take both within an hour. Black coffee is the best vehicle for administering these powders.—*Ex.*

